# Improving Heart Disease Risk Through Quality-Focused Diet Logging: Pre-Post Study of a Diet Quality Tracking App

**DOI:** 10.2196/21733

**Published:** 2020-12-23

**Authors:** Bum Chul Kwon, Courtland VanDam, Stephanie E Chiuve, Hyung Wook Choi, Paul Entler, Pang-Ning Tan, Jina Huh-Yoo

**Affiliations:** 1 IBM Research Cambridge, MA United States; 2 MIT Licoln Laboratory Lexington, MA United States; 3 AbbVie Inc Chicago, IL United States; 4 Division of Preventive Medicine, Department of Medicine Brigham and Women's Hospital and Harvard Medical School Boston, MA United States; 5 College of Computing and Informatics Drexel University Philadelphia, PA United States; 6 Sparrow Health System Lansing, MI United States; 7 Department of Computer Science and Engineering Michigan State University East Lansing, MI United States

**Keywords:** mHealth, diet monitoring, diet tracking, food tracking, CVD, heart disease risk, health risk communication, human-computer interaction, user study, mobile phone

## Abstract

**Background:**

Diet-tracking mobile apps have gained increased interest from both academic and clinical fields. However, quantity-focused diet tracking (eg, calorie counting) can be time-consuming and tedious, leading to unsustained adoption. Diet quality—focusing on high-quality dietary patterns rather than quantifying diet into calories—has shown effectiveness in improving heart disease risk. The Healthy Heart Score (HHS) predicts 20-year cardiovascular risks based on the consumption of foods from quality-focused food categories, rather than detailed serving sizes. No studies have examined how mobile health (mHealth) apps focusing on diet quality can bring promising results in health outcomes and ease of adoption.

**Objective:**

This study aims to design a mobile app to support the HHS-informed quality-focused dietary approach by enabling users to log simplified diet quality and view its real-time impact on future heart disease risks. Users were asked to log food categories that are the main predictors of the HHS. We measured the app’s feasibility and efficacy in improving individuals’ clinical and behavioral factors that affect future heart disease risks and app use.

**Methods:**

We recruited 38 participants who were overweight or obese with high heart disease risk and who used the app for 5 weeks and measured weight, blood sugar, blood pressure, HHS, and diet score (DS)—the measurement for diet quality—at baseline and week 5 of the intervention.

**Results:**

Most participants (30/38, 79%) used the app every week and showed significant improvements in DS (baseline: mean 1.31, SD 1.14; week 5: mean 2.36, SD 2.48; 2-tailed *t* test *t*_29_=−2.85; *P*=.008) and HHS (baseline: mean 22.94, SD 18.86; week 4: mean 22.15, SD 18.58; *t*_29_=2.41; *P*=.02) at week 5, although only 10 participants (10/38, 26%) checked their HHS risk scores more than once. Other outcomes, including weight, blood sugar, and blood pressure, did not show significant changes.

**Conclusions:**

Our study showed that our logging tool significantly improved dietary choices. Participants were not interested in seeing the HHS and perceived logging diet categories irrelevant to improving the HHS as important. We discuss the complexities of addressing health risks and quantity- versus quality-based health monitoring and incorporating secondary behavior change goals that matter to users when designing mHealth apps.

## Introduction

### Background

An increasing number of mobile apps have explored ways to monitor and improve health behavior efficiently and effectively [1-3]. Among these mobile health (mHealth) apps, diet monitoring is one of the most popular domains, as diabetes and obesity are shown to lead the top 2 fields producing revenue in the mHealth market [4]. A systematic review of mobile apps showed that mHealth apps on obesity and nutrition increased adherence to diet monitoring and effectively improved primary clinical outcomes, such as weight loss and maintenance of reduced blood glucose levels [1,5].

However, for effective dietary monitoring, ideally, users would need to quantify their food consumption down to the level of the number of grams of each nutrient [6]. Focusing on such a quantification of diet can bring several challenges. Food journaling can be *too much effort, time-consuming, or tedious* [7,8]. Food journaling with detailed entries can be challenging as users often might not remember or know what and how much they have eaten [9]. Users also feel that the dietary information in the database is unreliable, calories burnt seemed random and *did not line up* [10], and entering unhealthy food consumption in detail makes people feel guilty in general. As these barriers lead to limited engagement with diet-tracking apps, researchers have attempted lightweight approaches of diet tracking, and such attempts have been shown to be successful by providing users with a photo-based food-tracking app and encouraging them to track only one food type per day [11].

A 2018 study published in the *Journal of American Medical Association* showed the effectiveness of focusing on diet quality over quantity—with a focus on restricting low-quality foods, such as processed foods, added sugar, or refined grains—rather than calorie counting [12]. However, mobile apps on dietary monitoring focused on the quantification of diet (eg, calorie counting) and other health behaviors (eg, steps). This quantification approach does not necessarily address the needs of broader groups of individuals. Numeracy and literacy in general can be barriers. People show increased confusion around the serving size [13]; however, for these apps to work appropriately, it would require accurate calculations of these very nuanced behavior choices. For instance, one might have eaten a mixed salad, but the system needs to know how many grams of spinach versus carrots and which salad dressing were consumed to calculate accurate calories and nutritional content. Sophisticated, detailed, quantified tracking practices are not popular for all user groups [14]. Tracking detailed health information is a user burden, affecting sustained tracking behavior [8].

The effectiveness of mHealth includes seeing the effect of behavior change. The knowledge of risk level helps individuals understand how urgently they need to change their behavior. Individuals at higher risk are more motivated to change if they know that they are at high risk [15]. A mobile app allowing users to observe how their risks are affected by their day-to-day choices relating to health and wellness (eg, such as their choice of food that day) can greatly help in the prevention of chronic diseases. The awareness of heart disease risk is one of the most critical methods and strategies to change behavior. Numerous mobile apps have been designed to bring awareness directly or indirectly about heart disease [16,17]. However, these apps rarely show how lifestyle behavior changes related to risk factors—smoking, diet quality, or alcohol consumption—affect their outcomes to prevent heart disease [1,16-22]. Although understanding future risks increases motivation of individuals to change behavior, whether individuals will actually change their behavior is a more complicated, sophisticated problem to solve than just *getting the message across* [23].

### Objectives

Our goal was to design and test a mobile app that would help users focus on improving diet quality with the help of real-time feedback on future heart disease risk as a result of their diet quality patterns. In this way, we could increase individuals’ awareness of cardiovascular risks based on daily dietary choices. Thus, users can focus on the behavior that is present and immediate, rather than an uncertain future [24,25]. Users can log simplified categories that have a high-quality diet—for example, vegetables, fruits, and whole grains—to help them focus on the quality of food rather than the detailed nutritional value, calories, and quantity of food. Our research questions (RQs) are as follows:

- RQ 1: How feasible was logging diet quality?
- RQ 2: How feasible was communicating risk to motivate behavior change?
- RQ 3: How effective was the app in changing health outcomes?

Our study demonstrated that (1) monitoring simple diet quality can have a significant effect on dietary behavior change and (2) regardless of participants’ interest in heart disease risk, the app reduced the risk.

## Methods

### Study Design

We designed the app based on behavior change techniques (BCTs) [26]. We used focus groups to iteratively improve the paper prototypes and developed an Android-based app as a result. We then conducted a 5-week pre-post study with a follow-up at 2 weeks after the study to evaluate the app’s efficacy of clinical and behavioral outcome changes as well as app usage patterns.

### Focus Groups for App Development

**Figure 1:**
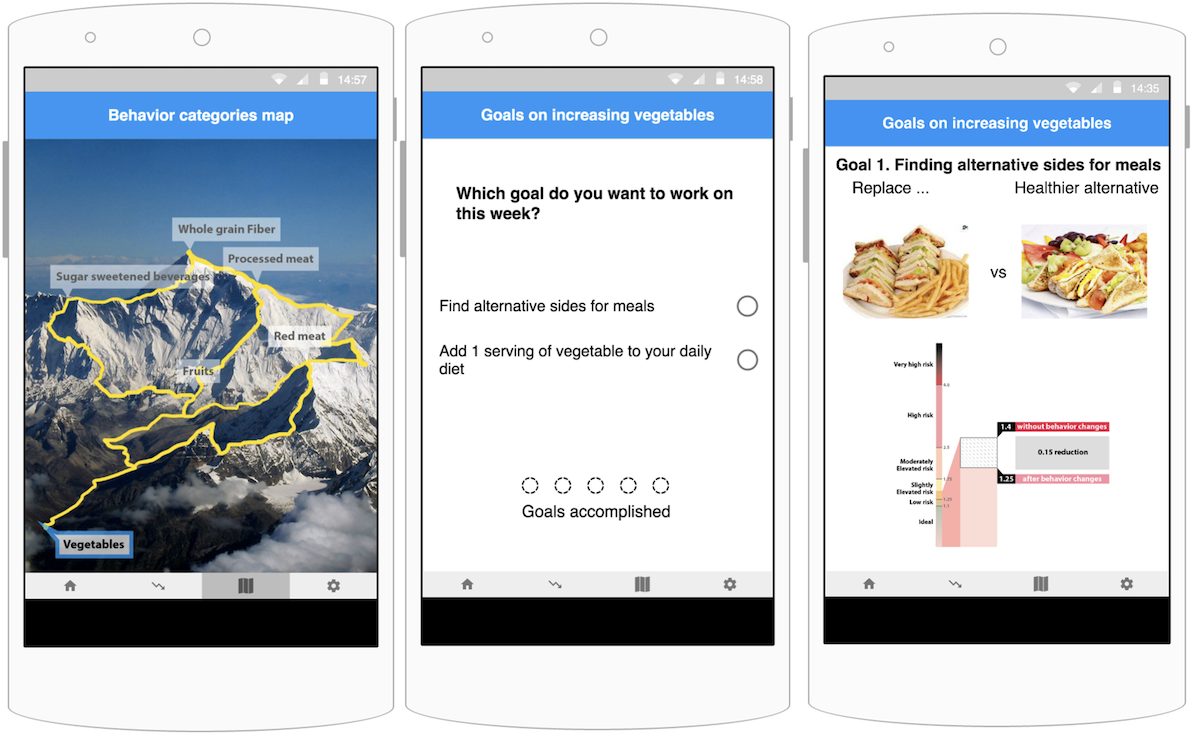
Screens from the prototype presented to the focus group. Users can select which goal to work on using the mountain climbing metaphor (left). As users accomplish the goals, they can unlock the next category of goals. Selecting a category on the behavior category map will direct the user to the goal selection screen (center). The screen on the right shows choice for sides and how future cardiovascular risks might differ, if the user were to repeat the behavior for a week.

We conducted 3 focus groups in a sequence (n=13, with 3-5 people for each group) to iteratively improve the initial digital paper prototype (Figure 1). The participants were at risk for heart disease and were recruited from a weight management clinic in the US Midwest. During the focus groups, the participants were presented with images from the initial prototype to test the usability and learnability of each screen (Figure 1). We revised the design iteratively based on the feedback. We then developed a mobile app on an Android platform.

### Final App Design

The BCT suggests 4 core components in designing an intervention: environmental contexts, goals, feedback and monitoring, and reinforcement. The app contains 5 screens: *main menu, profile, goals, meal calendar* (food logging screen), and *cardiovascular risk* (screen showing heart disease risk score). We designed the profile page to incorporate environmental context, the goals menu for users to personalize goals, meal calendar to log diet quality for feedback and monitoring, and cardiovascular risk screen to reinforce and reward positive diet change. A first-time user is directed to the profile screen to provide demographic information related to calculating their risk.

### Diet Quality and Healthy Heart Score

**Figure 2:**
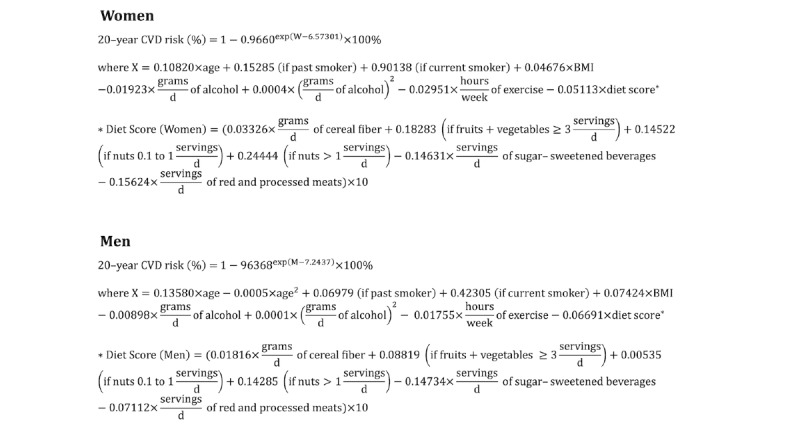
Description of the Healthy Heart Score and calculation of diet score for women and men.

The definition of high-quality diet in this study was based on the Healthy Heart Score (HHS), a risk score system for heart disease risk, developed at Harvard University (Figure 2) [15]. Among several heart disease risk models (eg, Framingham) [27], HHS is uniquely useful for middle-aged adults who do not have elevated clinical factors, such as high blood pressure or cholesterol, but may still be at high risk for developing cardiovascular disease (CVD). The HHS model builds on lifestyle factors, such as smoking status; level of physical activity; alcohol intake; and a diet score (DS) based on the consumption of fruits and vegetables, nuts, cereal fiber, sugar-sweetened beverages, and red and processed meats. HHS measures diet quality using the DS factor (Figure 2). A high DS indicates that the individual is eating more *healthy foods*, including fruits, vegetables, nuts, and white meat. Consumption of *unhealthy foods*, including red meat, processed meat, and sugary drinks, will lead to lower DS.

**Figure 3:**
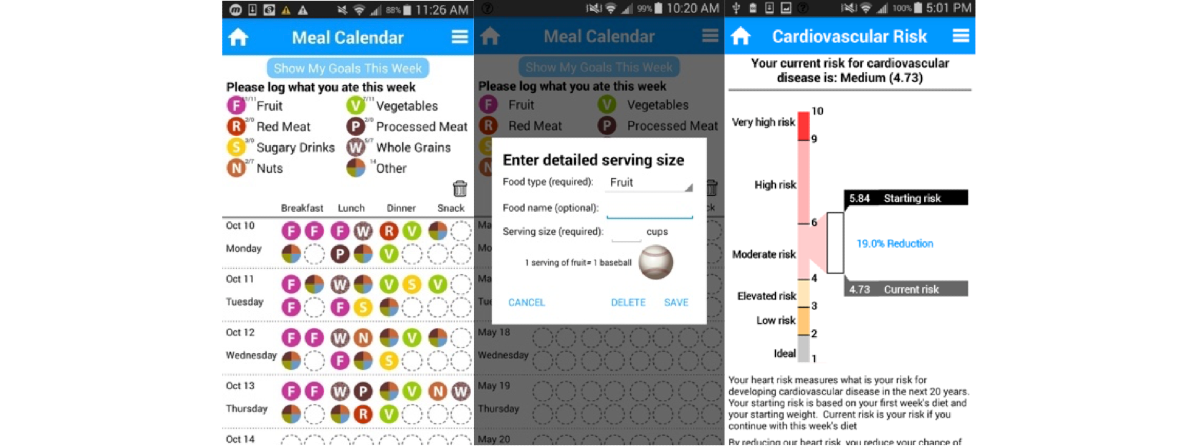
The left panel shows the meal calendar screen, where users can enter simple quality-oriented diet categories. The central panel shows options to add more details about the food, if the user desires. The right panel shows the screen that updates Healthy Heart Score as the user enters diet information.

In the diet-monitoring screen (Figure 3, left), users can enter up to 4 food categories for each meal they eat each day: breakfast, lunch, dinner, and snack. Following HHS, users can log the overall quality of diet through the 7 food category items noted by HHS: 4 *healthy categories*, that is, fruits, vegetables, whole grains, and nuts, and 3 *unhealthy categories*, that is, red meat, processed meat, and sugary drinks. The app also allowed the selection of other categories to log foods not included in the provided categories. The goals screen showed the default number of servings suggested for each food category. Users can either drag a food category icon, for example, fruit, to one of the meal slots, which counts as one serving of that category to that meal, or tap the calendar and work on the pop-up window to increase or decrease the number of servings and add the name of the food they consumed. The definition of a serving was not defined—any consumption counted as a serving, following the antiquantification approach. In the goal screen (Figure 3, center), the default suggestions on the intake amount of unhealthy food categories were set to 0 servings. The combined total for fruits and vegetables should be at least 3 servings per day or, equivalently, 21 servings per week.

### Future Cardiovascular Risk

The cardiovascular risk (Figure 3, right) screen shows the current HHS, the user’s calculated cardiovascular risk score in real time. The screen compares the risk at the time the user started using the app with the risk at the current week. In this risk screen, we rescaled the HHS to a range from 1 to 10 from its original unit, 0% to 100%, following the suggestion provided by the focus groups and in consultation with the expert who developed the HHS. The focus groups complained that the percentage was confusing—for example, it was unclear whether 50% meant 50% higher risk than others or half of the risk compared with others (or compared with the current status). In the rescaled scoring range, the ideal risk score for a healthy individual is between 1 and 2, and if one has a risk score of 9 or above, the person is 4 times or more likely to develop heart disease than an individual with a healthy lifestyle.

### Goals

At the beginning of each week, the app prompts users to set their goals and directs them to the goals screen. Users can press the goal icon of the food categories they want to actively work on. Users can deactivate a goal by tapping it again, and the goal will appear grayed out. If one of the goals for unhealthy categories is active, users will be notified on the meal calendar if they exceed the maximum number of servings stated by the goal. The goals screen includes a checkbox that shows whether the user has met the goal.

### Pre-Post Study: Recruitment and Procedures

The participants were recruited from a weight management clinic at a major hospital in the US Midwest. A research coordinator waited in the waiting room of the clinic with a recruitment flyer and screened interested potential participants for the following criteria: (1) aged above 18 years, (2) self-reported as overweight or obese, and (3) self-reported as at risk for diabetes. A total of 38 participants were enrolled to start the intervention between June and September 2016 for a 5-week period (denoted as week 0-4), with a follow-up meeting at 2 weeks after the end of week 4. The participants were asked to use the app for at least 6 days a week for the 5-week period of the study. The participants had the option to continue using the app until the follow-up meeting. Initially, the participants were asked to log their diet to establish a baseline. Starting at the beginning of week 1, the app started prompting the participants to set goals for each week based on HHS recommendations, either by keeping the default suggestion (ideal diet) or changing it to personalized goals.

**Figure 4:**
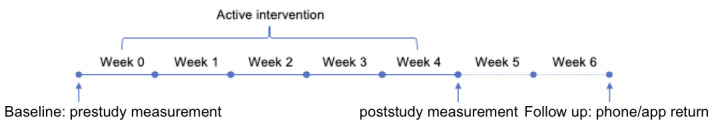
Timeline of prestudy and poststudy measurements and follow-up and the notation of the weeks.

At baseline and at the end of week 4, participants visited the clinic for a clinician to measure their weight and fasting blood sugar levels. At the end of week 4, participants were reminded that they were no longer required to use the app. In addition, an exit interview was held at the follow-up visit to discuss the participants’ experiences with the app. Figure 4 shows the study procedure.

All participants received cash compensations of up to US $50. Participants received partial or full compensation depending on how much they completed the following: 3 web-based surveys, measuring health outcomes twice, and using the app for at least 6 days a week during week 0 to 4. The app was provided to the participants in 2 ways. If the participants had an Android phone, the app was installed on their phones. Otherwise, the participants were provided with a Samsung Galaxy S3 phone, with the app installed, for the duration of the study. These participants were required to return their phones at the follow-up.

The study was approved with a full review by the institutional review board (IRB) of the institution that collected the data. All other researchers of this study were given access to deidentified data with the permission of the IRB.

### RQs and Analyses

We wanted to answer 3 RQs regarding the feasibility of logging diet quality, motivating behavior change through feedback on future heart disease risks, and the app’s efficacy of behavior change.

#### RQ 1: How Feasible Was Logging Diet Quality?

We recorded and analyzed the time, frequency, and screen activity of the participants’ tapping events on the app. We analyzed how often participants went to each screen and which food categories were logged over time. We also analyzed user logs of food names to understand diet logging behavior.

#### RQ2: How Feasible Was Communicating Risk to Motivate Behavior Change?

We analyzed the participants’ usage of the risk screen. We then tested the correlation between the participants’ usage of the screen and HHS.

#### RQ3: How Effective Was the App in Changing Health Outcomes?

We conducted a paired sample *t* test to compare the outcome changes in diet quality, HHS, and in-clinic measurements (weight and fasting blood glucose levels) between pretest (at the beginning of week 0) and posttest (at the end of week 4) measurements. We used the statsmodels package in Python to run the analysis.

## Results

### Overview

In this section, we first report the demographic information of the participants and the recruitment outcome. We then report results on diet quality, risk score checking, health outcomes and DS, and the association between app use and DS.

### Participants and App Use

A total of 84% (32/38) of the recruited participants completed the follow-up. Table 1 summarizes the demographic information of the participants. Overall, 58% (22/38) of participants used the study phones provided and the rest used their own phones. In total, 8% (3/38) of the participants who were Android phone users stopped using the app and stopped responding to the researchers. Another participant (1/38, 3%) withdrew as the app was too cumbersome. Furthermore, 5% (2/38) other participants withdrew as they decided that the study no longer applied to them. The remaining 32 participants (female: 26/32, 81%; age (years): mean 57.48, SD 11.85) who completed the study had a wide range of age, weight, smoking status, and experience of using a smartphone. A total of 3% (1/32) of the participants were smokers, 38% (12/32) were former smokers, and 59% (19/32) were nonsmokers. A total of 53% (17/32) of participants were diagnosed with diabetes. Overall, 13% (4/32) of participants were overweight (BMI between 25 and 29.9 kg/m^2^) and the remaining 88% (28/32) of participants were obese (BMI≥30) [28].

**Table 1:** Demographic information of participants.

Measure		Value	
Participants who completed follow-up^a^, n (%)		32 (85)	
Participants who used the provided phones^a^, n (%)		22 (58)	
Age of participants who completed follow-up^b^, years, mean (SD)		57.48 (11.85)	
Female participants^b^, n (%)		26 (81)	
**Participants who smoked, n (%)**			
	Current		1 (3)
	Former		12(38)
	Nonsmoker		19 (59)
Participants with diabetes^b^, n (%)		17 (53)	
**Weight of participants, n (%)**			
	Overweight		4 (13)
	Obese		28 (88)

^a^The total number of participants is 38.

^b^The total number of participants who enrolled is 32.

### RQ 1: How Feasible Was Logging Diet Quality?

#### App Use

**Figure 5:**
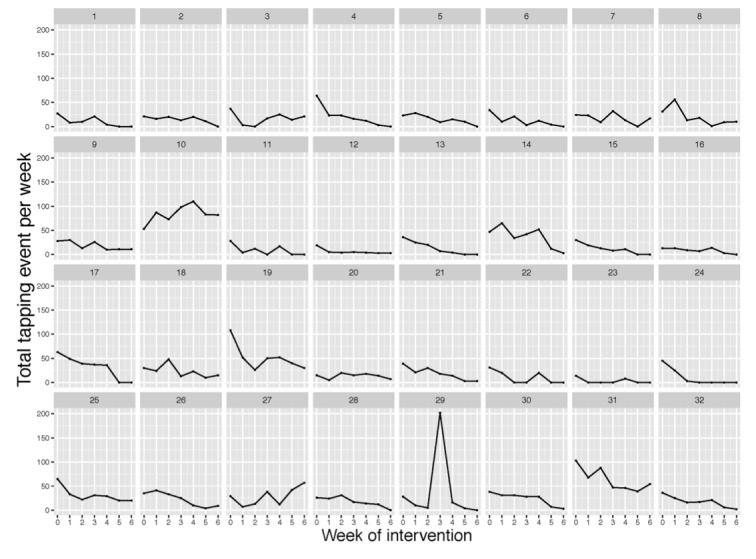
Each small graph shows each participant’s total number of tapping events over the entire 7-week period, including the 2-week follow-up (between the baseline and follow-up). The x-axis shows the week of intervention (0 indicating the frequency accumulated between the baseline and at the end of week 0). The y-axis shows the total number of tappings for each week. A total of 27 participants visited the screen approximately every week for the intervention duration (weeks 0-4).

During the active intervention when the participants were required to use the app (baseline to week 4), participants *tapped on the app*, that is, clicked to view contents or to make food entries, 27 times on average (SD 25.6) per week. After week 4 and until follow-up, participants tapped on the app 11 times on average (SD 18.3) per week. Figure 5 shows each participant’s overall app use over the weeks.

#### Diet Logging

**Figure 6:**
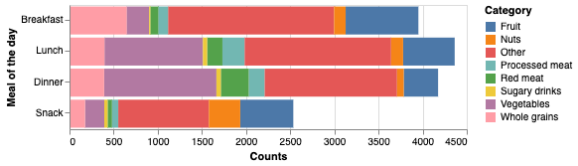
All 32 participants’ logging per food category and for which meal of the day the logging occurred during the active intervention period (between baseline and the end of week 4).

Overall, 88% (28/32) of participants logged food consumption data every week between the baseline and the end of week 4, at least once a week. About half of the participants logged food consumption data approximately every day. As seen in Figure 6, during the active weeks, the 32 participants altogether logged *other* the most (6066/15,025, 40.37%), followed by *vegetables* (2857/15,025, 19.01%), *fruit* (2398/15,025, 15.96%), *whole grains* (1614/15,025, 10.74%), *nuts* (698/15,025, 4.65%), *red meat* (627/15,025, 4.17%), *processed meat* (614/15,025, 4.09%), and *sugary drinks* (151/15,025, 1.00%). Figure 6 shows when these food categories were logged to meal slots during the course of the day—breakfast, lunch, dinner, or snack. Fruits and whole grains were logged proportionally larger during breakfast meals than during other meals, and vegetables were logged proportionally larger during lunch and dinner meal times. *Other* categories were logged equally over all meal slots.

Participants entered a qualitative description of the food in the *food name* field for 38.14% (5730/15,025) of all diet logging instances. Overall, 48.87% (2800/5730) of these instances were entered when logging to the other category, 18.00% (504/2800) for vegetables, 15.00% (420/2800) for fruit, 8.00% (224/2800) for whole grains, 4.00% (112/2800) for nuts, 3.00% (84/2800) for red meat, 3.00% (84/2800) for processed meat, and 0.68% (19/2800) for sugary drinks. For all categories except other, participants gave a sample description of the food category they entered. For instance, the fruit category included descriptions such as *strawberries* or *grilled fruit salad* and the vegetable category included *arugula* or *grilled squash and zucchini with lemon and olive oil*.

When participants entered *other*, 98.89% (2769/2800) included detailed descriptions of the food. The qualitative analysis of these descriptions, together with the exit interviews, revealed several reasons for why the food was logged as *other*. The given food categories did not capture all the food categories the participants attempted to log, such as their current dietary goals (eg, to reduce dairy). The participants were instructed to only log what is related to heart disease risks, but they still captured other categories not affecting healthy heart risk, including dairy, dessert, or other protein foods (eg, 338/2769, 12.20% were proteins such as eggs, tofu, and beans and 584/2769, 21.09% were dairy such as milk, cheese, and Greek yogurt). Participants also logged foods as belonging to *other* category when the food was a mix of various food categories, which might have been difficult to capture in 1 or 2 food categories (eg, California roll, sandwich). A total of 0.69% (19/2769) showed red meat food, such as pork, and vegetables being logged as *other*, showing how the users might have been confused on what food categories these foods belonged to. Even though pork was red meat, the fact that it was logged as *other* matched with the exit interview content that the participants considered pork as white meat.

#### Risk Screen

**Figure 7:**
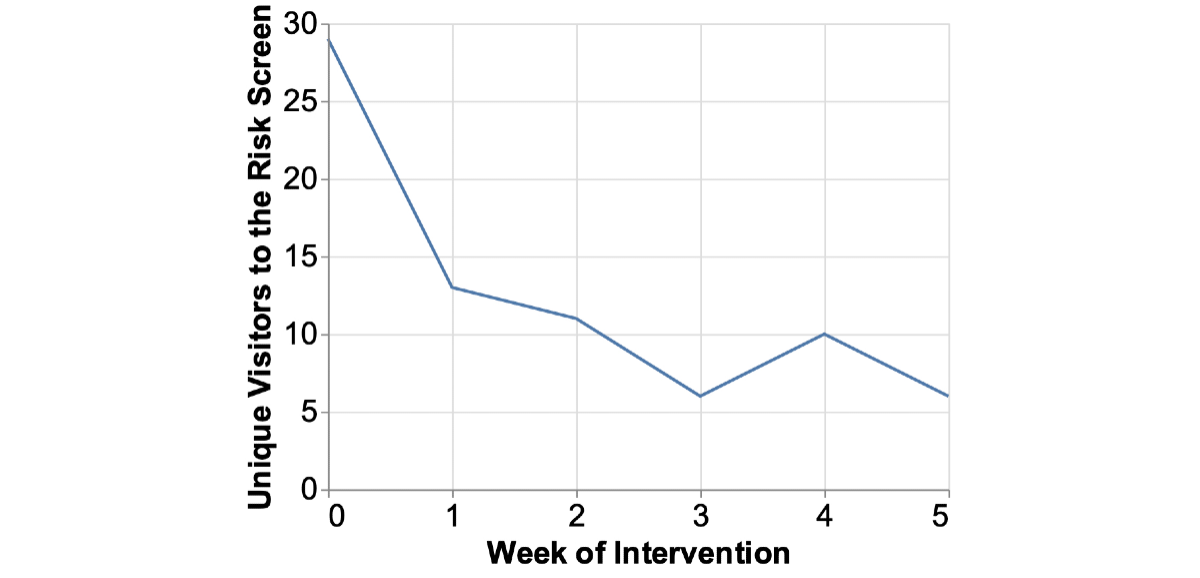
This figure shows the participants’ use of the risk screen (loading frequency) over the weeks. A total of 29 of 32 participants checked their risks during week 1, and then, only a few checked again at week 4 (n=10). Most participants did not return to the risk screen to recheck it after the baseline.

As Figure 7 shows, at baseline, most participants checked their risk scores (n=29). However, in the following weeks after the baseline, most participants did not return to the risk screen to view the changes in their HHS. A total of 41% (13/32) people checked the risk screen in week 1, 34% (11/32) in week 2, 19% (6/32) in week 3, 31% (10/32) in week 4, and 19% (6/32) in week 5 until follow-up.

### RQ 3: How Effective Was the App in Changing Health Outcomes?

#### Health Outcomes

##### DS

**Figure 8:**
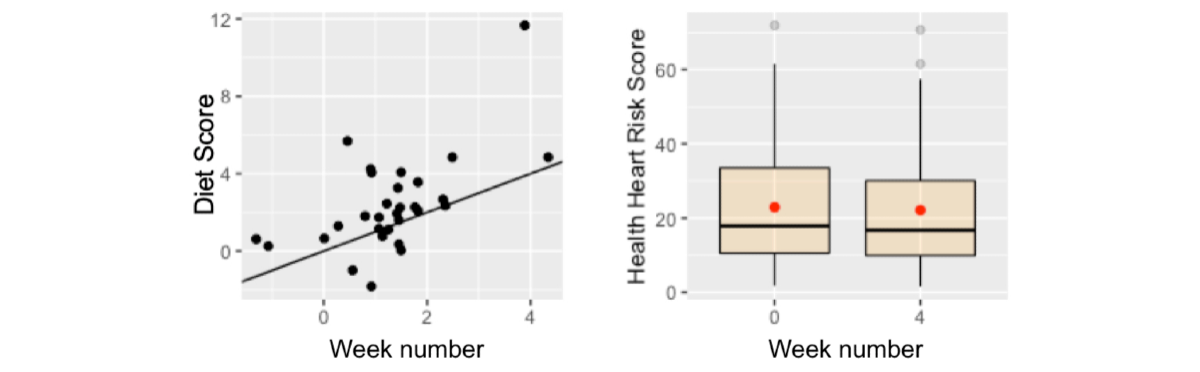
Changes in diet score (left) and Healthy Heart Score (right) between prestudy and poststudy measurements of the participants.

All but 6% (2/32) of participants logged their diet during the active intervention. Among the 94% (30/32) of participants who logged their diet during the active intervention, the DS showed a significant difference between baseline (mean 1.31, SD 1.14) and posttest during week 4 (mean 2.36, SD 2.48; t_29_=−2.85; *P*=.008; Figure 8).

##### HHS

HHS also showed significant difference between baseline (mean 22.94, SD 18.86) and posttest measurements at the end of week 4 (mean 22.15, SD 18.58; t_29_=2.41; *P*=.02).

There was no statistical association between food logging frequency and the 3 measures—DS, risk, and BMI.

##### In-Clinic Measurements

Weight (lbs) did not show a significant difference between pretest (mean 241.7, SD 61.17) and posttest (mean 242.6, SD 61.9) measurements, t_29_=−1.043, *P*=.31. Blood sugar level (mg/dL) also did not show a significant difference between the pretest (mean 130.2, SD 76.62) and posttest (mean 123.3, SD 48.8) measurements, t_28_=−0.95, *P*=.35.

## Discussion

### Principal Findings

The study showed feasibility for logging diet quality (RQ 1) but not for communicating risk (RQ 2). However, the app was effective in changing health outcomes (RQ 3), showing that logging simplified diet quality significantly improved dietary scores and future cardiovascular risk scores. The following are the key takeaways:

- The study showed no association between the frequency of logging and improved dietary scores, showing the importance of separating frequency of use in measuring health outcomes.
- The participants were not interested in monitoring the risk scores, but they still significantly decreased their risk scores by focusing on the target behavior. This finding has implications for health risk communication in mHealth app design.
- The study showed that users mostly logged irrelevant dietary behaviors to the target behavior. This finding shows the need to balance reducing monitoring items for efficiency versus what matters to users to support user experience.

### Opportunities and Challenges of Quality-Focused Diet Monitoring

Previous literature shows that logging diet is highly associated with improved diet [29]. At the same time, studies have shown that not all users can benefit from sophisticated diet logging apps. Users often find diet logging a tedious, cumbersome activity, which leads to abandonment [7]. In addition, people do not always accurately estimate food proportions and nutritional contents [13]. Automated techniques, including calorie calculations and artificial intelligence–based food detection, can reduce such user burden in detailed diet logging [30-32]. However, these methods are still limited and error prone, which leads to increased user frustration and abandonment.

To address this gap, we implemented the HHS [15] into a mobile app, which simplified the diet-monitoring process to focus on improving diet quality over quantity. This incorporates a lenient approach toward logging food proportion and nutritional details in calculating the risk. By allowing users to focus on logging simplified diet quality that does not require logging detailed nutritional and caloric breakdown of each meal and focusing on whether a gross food group was consumed (fruits and vegetables), we showed that users steadily used the app even after the required period, for which they were not incentivized to use it. One participant asked if they could continue using the app even after the study had completed.

To our knowledge, our study is the first to investigate the feasibility of an mHealth solution for logging diet quality. Existing diet-monitoring apps have focused on logging the granular details of diet at a nutritional level [1]. Our findings on participants’ use of our app are comparable with the user groups of other studies that showed a successful adoption of an mHealth tool. Individuals who complied with the study protocol regularly entered their food consumption details between 2 and 4 times a day [6,7,33].

At the same time, the study showed no association between frequency of use and increase in DS. This finding shows the need to separate quantitative measures of use from health outcomes. This implication aligns with discussions about whether sustained use of an mHealth app is positive or not—discontinuing to use an app might mean that the user no longer needs the app as they have achieved the health goal or have become more independent [34]. Furthermore, there can be other factors that influence the participants’ healthier choices. According to Achananuparp et al [35], food journalers do not necessarily eat healthier food than others. Healthy eating behaviors are often affected by other sociodemographic factors, such as gender and regions of residence.

One challenge we faced in logging diet quality was that, even at the gross level of food categories, some participants were confused regarding categorizing food to the right categories (eg, confused pork as white meat, avocado as not being vegetable). Studies report that people struggle with journaling food regularly as the task is often time-consuming and laborious [8]. Therefore, it is desirable to implement a positive incentive for food journaling and to remove barriers for users. One idea is to help users go over their health management progress with their doctors, as Kim et al [33] tested in their study. We can implement photo-based food journaling with some automatic support to identify the category and to measure the portion of food [36]. Such photos can be distributed via social media, which can play a role in providing social support and encouragement for users as well.

### Implications for Health Risk Communication in mHealth Design

The initial goal of this app was to increase individuals’ awareness of cardiovascular risks based on daily dietary choices. We expected that users would check their risk scores as they changed their dietary patterns to understand how their risks were impacted by their dietary choices, thus making behavioral changes. However, although logging the diet quality was positively accepted by the participants, the participants rarely visited the risk screen during the study period. The participants mainly visited the risk screen at the very beginning to check their initial risk score, and a few came back for a second check after a week, and most did not return. The follow-up interview revealed that the participants noted that their scores did not seem to visibly change, so they did not think to check more often. At the same time, the HHS results showed that the participants still significantly improved their HHS at week 4. The HHS is associated with many clinical disease risks, such as diabetes mellitus, hypertension, and hypercholesterolemia [37]. In other words, studies report that patients with higher HHS show higher incidences of such diseases [37,38]. When we converted the HHS to 20-year CVD risk, the participants’ initial risk resulted in 100%. This is because we recruited participants at high risk for heart disease due to their overweight or obesity condition. An HHS higher than 13 is calculated as 100% for the 20-year CVD risk. Accordingly, the change in the HHS from 22.94 to 22.15 will not decrease the CVD risk to 100%. Even a slight reduction of the HHS can decrease the CVD risks at this point. Despite the limited samples and duration, this study shows that the participants on average reduced their HHS by 0.78%, which is encouraging. An unanticipated design challenge that emerged in this study was regarding visualizing risks for those who were already well above the risk and for whom the risk would have remained at 100% despite any behavior changes they made. One idea is to augment a forecasting trajectory to the risk score, focusing on the changes toward reducing risks. The prediction can be designed so that it adjusts more sensitively to users’ recent efforts for more motivation.

Communicating future risks is known to alert and motivate people to change behavior [39-41]. At the same time, if risk is too distant in the future, people may feel the risk is irrelevant, making it difficult to make behavior changes [42,43]. This study showed that the participants were initially motivated by their risk score; however, the behavior change was not related to their checking of the risk score over time. Although users did not check their risk scores, they decreased their overall risk scores. This finding has implications for the role of health risk communication in consumers using mHealth apps, in which continuous monitoring is the strength. The risk scores can serve as initial motivation to set up goals; however, users could focus on monitoring and improving the target risk behavior (in this case, diet quality), and the improvement with the risk can be a positive side effect.

### Implications of Other in Monitoring Apps

The findings on the largest logging activity of *other* food categories provided implications for balancing between simplification and accommodation of users’ *other* needs. The HHS discourages or encourages certain food categories to be consumed. This instruction—to focus on improving consumption of certain food categories—was reassured to the participants during the instruction. In the app design, we also specifically allowed users to only log the relevant food categories to improve the HHS. However, most diet logs were under *other*, where it included irrelevant food categories, such as dairy. According to the follow-up interview, this came from having a concurrent diet goal of participants on their own. When designing a monitoring app to improve health behavior, one needs to consider the gap between the chosen clinical approach and individuals’ concurrent goals and considerations. Although simplifying the design to only monitor necessary information can improve efficiency and reduce user burden, this design approach might lose incorporating users’ concurrent needs and focus. One should not consider what matters to users as *other* because it is considered irrelevant to what we designed as a target goal.

### Limitations

This study did not include a control group, and the duration was only 5 weeks, which is not sufficient to show true behavior change. The data did not include information on whether the participants discontinued checking risk scores because of the lack of usability (eg, legibility of the visualization) or their disinterest in risks.

### Conclusions

Our study showed the feasibility and efficacy of simplified diet quality monitoring in an mHealth app. Future work should further test the app’s efficacy with a larger, focused population that is disinterested in using existing quantity-focused monitoring apps. Despite some known limitations on research design and duration, the findings provided significant contributions to understand the implications on the opportunities and challenges in designing a simplified, diet quality–focused monitoring app and how health risk communication can be effectively integrated into an mHealth design. The study also sheds light on finding the balance between affording users to focus on simplified target behavior, reducing user burden versus further incorporating what matters to users in designing a health monitoring app.

## Abbreviations

BCT: behavior change technique
CVD: cardiovascular disease
DS: diet score
HHS: Healthy Heart Score
IRB: institutional review board
mHealth: mobile health
RQ: research question
